# Brazilian Green Propolis Carried in Lipid-Based Nanostructures: A Potent Adjuvant Therapy to Non-Surgical Periodontal Treatment in the Management of Experimental Periodontitis

**DOI:** 10.3390/biomedicines13071643

**Published:** 2025-07-04

**Authors:** Glauco Rodrigues Carmo Silveira, Vinícius Franzão Ganzaroli, Luan Felipe Toro, Leandro Lemes da Costa, Rodrigo Isaias Lopes Pereira, André Bueno da Silva, Iasmin Rosane Silva Ferreira, João Martins de Mello-Neto, Valdir Gouveia Garcia, Letícia Helena Theodoro, Priscyla Daniely Marcato, Edilson Ervolino

**Affiliations:** 1 Department of Basic Sciences, School of Dentistry, São Paulo State University (UNESP), Araçatuba 16015-050, SP, Brazil; glauco.silveira@unesp.br (G.R.C.S.); vinicius.ganzaroli@unesp.br (V.F.G.); luan.toro@unesp.br (L.F.T.); leandro.lemes@unesp.br (L.L.d.C.); rodrigo.l.pereira@unesp.br (R.I.L.P.); andre.b.silva@unesp.br (A.B.d.S.); 2 Department of Diagnostic and Surgery, School of Dentistry, São Paulo State University (UNESP), Araçatuba 16015-050, SP, Brazil; leticia.theodoro@unesp.br; 3 Institute of Biosciences, São Paulo State University (UNESP), Botucatu 18618-000, SP, Brazil; 4 Marília Medical School (FAMEMA), Marília 17519-030, SP, Brazil; 5 Department of Pharmaceutical Sciences, GNanoBio, School of Pharmaceutical Sciences, University of São Paulo (USP), Ribeirão Preto 14040-903, SP, Brazil; iasminferreira@usp.br (I.R.S.F.); pmarcato@fcfrp.usp.br (P.D.M.); 6 College of Medicine and Dentistry, James Cook University, Cairns, QLD 4870, Australia; joao.martinsdemelloneto@jcu.edu.au; 7 Latin American Institute of Dental Research and Education (ILAPEO), Curitiba 80810-030, PR, Brazil; valdir.garcia@unesp.br

**Keywords:** green propolis, lipid-based nanostructures, periodontitis, scaling and root planing, rat

## Abstract

**Objective**: This study aimed to evaluate the effects of local use of Brazilian Green Propolis (BGP), either as an ethanolic extract (the most common formulation) or incorporated into lipid-based nanostructures, as an adjuvant therapy for non-surgical periodontal treatment in managing experimental periodontitis (EP) in ovariectomized rats. **Methods**: Fifty-six female Wistar rats underwent bilateral ovariectomies. After 10 weeks, a cotton ligature was placed around the lower first molar and remained in place for two weeks to induce EP. The ligature was removed, and the rats were randomly assigned in the groups NLT (*n* = 14), SRP (*n* = 14), SRP-BGPee (*n* = 14), and SRP-BGPlns (*n* = 14). In the NLT group, no local treatment was performed. The SRP group received scaling and root planing (SRP), along with irrigation using a physiological saline solution. The SRP-BGPee group underwent SRP and irrigation with ethanolic extract of BGP. The SRP-BGPlns group underwent SRP and irrigation with BGP-loaded lipid nanostructure (BGPlns). Each group received one SRP session followed by four irrigation sessions with the specified solutions, which were conducted immediately after SRP and subsequently after 2, 4, and 6 days. Euthanasia was performed at 7 and 28 days following the removal of the ligatures. The hemimandibles were processed for the following analyses: microtomographic analysis; histological analysis; histometric analysis of the percentage of bone tissue in the furcation region (PBT); and immunohistochemical analysis for tartrate-resistant acid phosphatase activity (TRAP), transforming growth factor beta 1 (TGFβ1), and osteocalcin (OCN). **Results**: The SRP-BGPlns group demonstrated superior periodontal tissue repair, reduced alveolar bone loss, fewer TRAP-positive cells (at 7 days), and higher levels of immunolabeling for TGFβ1 (at both 7 and 28 days) and OCN (at 28 days) compared to the other experimental groups. **Conclusions**: The irrigation with BGP is an effective adjuvant therapy for non-surgical periodontal treatment in managing EP in ovariectomized rats. Its application in lipid-based nanostructures proved to be more effective than the ethanolic extract form.

## 1. Introduction

Periodontitis is a multifactorial chronic inflammatory disease associated with dysbiotic biofilm. It is characterized by the progressive destruction of the dental attachment apparatus [1,2,3] and is one of the main causes of tooth loss in adults [4]. Several factors can contribute to the severity of periodontitis and its progression, with the most significant being poor oral hygiene, genetic predisposition, underlying health conditions like diabetes, and lifestyle choices such as smoking [4,5]. In addition, the deficiency of ovarian hormones, particularly estrogen, is among the factors that increase the severity of periodontitis [6]. Experimental in vivo studies have shown that ovariectomy exacerbates the periodontal inflammatory condition and leads to increased alveolar bone loss in experimental periodontitis (EP) [7,8,9,10,11]. Corroborating clinical studies have shown that postmenopausal depletion, particularly of estrogen, leads to osteopenia/osteoporosis, which impacts the severity of periodontitis [12,13,14,15,16].

Non-surgical periodontal treatment, scaling and root planing (SRP), is the gold standard initial therapy to treat periodontitis, along with oral hygiene instructions [17,18,19]. While SRP yields satisfactory results for most patients, it does not entirely eliminate periodontopathogenic microorganisms, particularly in areas that are difficult to access and clean. Therefore, adjunctive therapies alongside SRP can improve the effectiveness of periodontal treatment by complementing its effects [20,21]. It is important to recognize that after SRP, the periodontal tissues enter a repair process. However, if the severity of periodontitis is worsened by patient-related factors, the treatment response may be compromised, leading to suboptimal results [4,5]. In these cases, adjunctive therapies may be beneficial, particularly those that offer antimicrobial effects, modulate the immune response, and simultaneously stimulate tissue repair [20,21].

The properties of propolis suggest it could serve as a potential adjuvant treatment for non-surgical periodontal therapy in managing periodontitis. Propolis is a resinous substance that bees collect from various parts of plants. Its composition varies based on factors such as geographic origin, bee species, and botanical source [22,23,24,25]. Generally, it contains a complex array of bioactive substances, with flavonoids, phenols, and terpenoids being notable for their anti-inflammatory, immunomodulatory, antioxidant, and antimicrobial properties [26,27,28,29]. Particularly with regard to Brazilian Green Propolis (BGP), it is produced by *Apis mellifera* bees, predominantly from a plant common in the southeastern region of Brazil, *Baccharis dracunculifolia*. The complex chemical composition of this type of propolis, particularly rich in phenolic compounds such as artepillin C or baccharin [30,31], gives it a greenish hue. In vitro studies have demonstrated the significant effects of propolis extract on periodontopathogenic microorganisms [32,33,34]. In addition, experimental in vivo studies have shown that the local application of propolis extract, including BGP, is an effective adjuvant therapy for SRP in the management of EP [35,36]. Some clinical studies in humans have demonstrated that SRP, combined with irrigation using propolis extract, including BGP, is more effective in treating periodontitis [37,38,39,40,41,42]. Although it has very positive effects, the use of propolis in the form of an extract has limitations, such as low bioavailability, reduced absorption, and poor transcellular efflux [43,44,45]. Additionally, the type of extract may vary in quality regarding its chemical composition, particularly with propolis. The most commonly used formulation is ethanolic propolis extract, one of the most effective in preserving the active substances in its composition, albeit with some limitations, such as the use of alcohol, low water solubility, potential mucosal irritation, and low stability, which may restrict its application [46,47].

Lipid-based nanostructures have emerged as a promising option for drug delivery systems, particularly for natural active ingredients such as propolis. The high surface area-to-volume ratio of nanostructures enables significant loading of active ingredients while also enhancing solubility, bioavailability, stability, resistance to degradation, diffusibility, and permeability through biological barriers [48,49,50,51]. One cost-effective lipid-based nanostructure for propolis is oil-in-water (O/W) microemulsion, which can generate lipid nanoparticles. This type of nanostructure can incorporate all the aforementioned properties, and it does not require expensive equipment, techniques, or reagents that would significantly increase production costs. In O/W microemulsions, water and oil—two immiscible phases—are combined in the presence of a surfactant and a co-surfactant to form oil droplets dispersed in water, stabilized by interfacial surfactant layers. These droplets typically have diameters smaller than 200 nm [52,53,54,55].

The use of propolis in extract form has shown beneficial effects in managing periodontitis. However, this type of formulation can limit its biological efficacy. Lipid-based nanostructures have the potential to overcome these limitations and enhance the biological effects of natural active ingredients, such as BGP. The present study aimed to evaluate the effects of local application of BGP, either as an ethanolic extract (the most commonly used form) or incorporated into lipid-based nanostructures (O/W microemulsion), as an adjuvant therapy to SRP in the management of EP in ovariectomized rats.

## 2. Materials and Methods

### 2.1. Preparation of Ethanolic Extract of BGP and BGPlns

The BGP extract used in this study was provided by the APISVIDA company (São Paulo, Brazil). Thirty grams of BGP was combined with 70% ethanol (Synth^®^, São Paulo, Brazil), with the final volume adjusted to 100 mL. The mixture was protected from light and subjected to moderate agitation for one week. The main phytochemical compounds present in the BGP extract were identified by HPLC-DAD (λ = 275 nm). This consisted of the ethanolic extract of BGP used in the SRP-BGPee group.

For the BGP nanoencapsulation in lipid-based nanostructures, the ethanolic extract of BGP was filtered and dried by lyophilization. In order to achieve a high concentration of solids in these formulations without the use of ethanol, O/W microemulsion was prepared, composed of linseed oil (Croda, São Paulo, Brazil) and polyoxyl 15 hydrostearate (BASF, São Paulo, Brazil) at a 1:10 weight ratio, BGP extract (110 mg/mL), and purified water to complete a final volume of 20 mL. The aqueous phase, containing water with surfactant, was poured into the oily phase, composed of linseed oil and BGP extract, under magnetic agitation for 8 h. This consisted of the BGP-loaded lipid nanostructure (BGPlns) used in the SRP-BGPlns group.

The evaluations of the size, polydispersity index (PDI), and zeta potential (ZP) of the BGPlns were performed by Dynamic Light Scattering (DLS) using a Zetasizer NanoZS90 (Malvern Panalytical Ltd., Malvern, UK). All measurements were carried out using samples diluted in 1 mM KCl (Synth^®^) solution.

The encapsulation efficiency of biomarkers of BGP (artepillin C and baccharin) in the lipid nanostructures was determined by an indirect method. For this, the dispersion of BGPlns was centrifuged at 5000× *g* using a Microcon ultrafiltration system with a filtration membrane having a molar mass cutoff of 10,000 g/mol (Millipore^®^, Burlington, MA, USA).

The filtrate was then quantified using ultra-efficiency liquid chromatography coupled to mass spectrometry, with a Single Quadrupole detector (SQ Detector 2, Waters^®^, Milford, MA, USA) with negative electrospray ionization (ESI-). The encapsulation efficiency was calculated using the following equation: EE (%) = {[artepillin C or baccharin]_initial_ − [artepillin C or baccharin]_filtrate_)/[artepillin C or baccharin]_initial_} × 100.

The JEM-100 CXII (JEOL, Tokyo, Japan) transmission electron microscope was used to assess the morphological analysis of the lipid nanostructures. BGPlns dispersion was deposited onto 200-mesh copper grids. A 1% uranyl acetate (Sigma-Aldrich, St. Loius, MO, USA) solution was then applied, followed by allowing the sample to dry at ambient temperature prior to analysis.

### 2.2. Animals, Ethical Aspects, and Sample Calculation

Fifty-six female Wistar rats (Rattus norvegicus) aged 6 months, with an average weight of 300–350 g, were used in this study. The rats were sourced from the Araçatuba School of Dentistry Animal Resource Centre, São Paulo State University “Júlio de Mesquita Filho” (FOA-UNESP). They were housed in the Animal Centre of the Department of Basic Sciences (Histology and Embryology) of FOA-UNESP. They were kept under the following conditions: 12 h light/12 h dark cycle; room temperature of 22 ± 2 °C; ventilation and exhaust system allowing 20 air changes per hour; relative humidity of approximately 55 ± 5%; and housing in plastic cages with a maximum of 4 animals per cage, with water and food available ad libitum. The experimental procedures followed the standards set by the National Council for the Control of Animal Experimentation (CONCEA), are in accordance with the ARRIVE guidelines (https://arriveguidelines.org, accessed on 20 June 2025), and the experimental protocol was approved by the Ethics Committee on the Use of Animals (CEUA) of FOA-UNESP (#593-2020).

Bioestat 5.3 software (https://www.mamiraua.org.br/pt-br/downloads/programas/bioestat-versao-53; Instituto Mamiruá, Manaus, Brazil, accessed on 28 October 2021) was used to calculate the sample size. The sample size was calculated to ensure a power greater than 80% (α of 5%; type B error of 20%). It was determined that seven repetitions would be the minimum number required per treatment.

### 2.3. Experimental Procedures Performed on Animals

Each animal was assigned a unique numerical identifier from 1 to 56. A table containing the randomized distribution of the numbered animals in the different experimental groups was generated using Minitab^®^ 17 software (Minitab Inc., State College, PA, USA). It was not necessary to exclude any animals in the present study.

To perform ovariectomy, installation and removal of the cotton ligature, execution of local treatments, and euthanasia, the rats were anaesthetized intramuscularly using the combination of ketamine hydrochloride (80 mg/kg; Syntec, São Paulo, Brazil) and xylazine hydrochloride (6 mg/kg; Syntec, São Paulo, Brazil).

**Figure 1:**
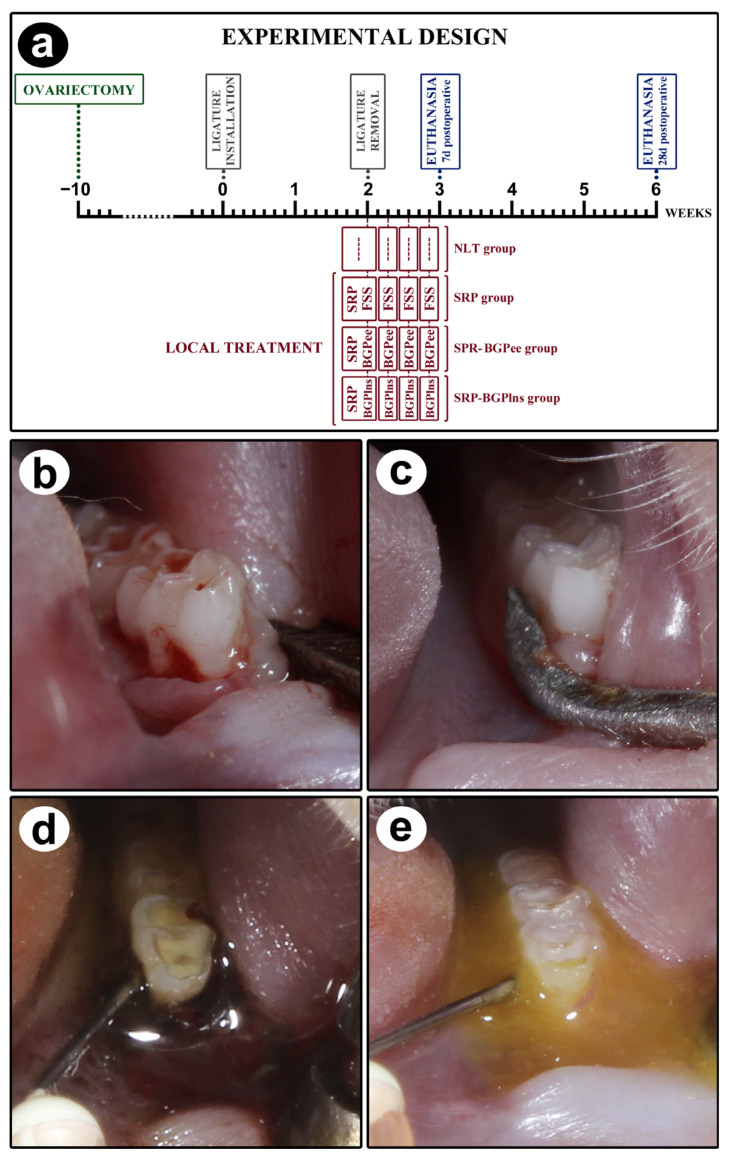
Study design. (**a**) Scheme depicting the procedures performed over time in NLT, SPR, SPR-BGPee, and SRP-BGPlns groups. (**b**) Clinical aspect after the removal of the ligature, which was kept for two weeks. (**c**) SRP procedure. (**d**) Local irrigation with ethanolic extract of BGP after SRP. (**e**) Local irrigation with BGPlns after SRP.

The scheme depicting the experimental procedures performed over time is shown in Figure 1a.

Ten weeks before the experiment began, all rats underwent ovariectomy. The abdominal area was subjected to trichotomy and asepsis with 10% povidone-iodine. Bilateral incisions were made to expose and remove the ovaries, the uterine tubes were reposited, and the incisions were sutured. Nine weeks after the ovariectomy, vaginal smears were collected from all rats between 7:00 and 9:00 a.m. for 7 days, according to the technique of Long and Evans (1922) [56], and analyzed fresh under an optical microscope to confirm the effectiveness of the ovariectomy. Confirmation of persistent diestrus allowed these rats to proceed to the other stages of the experiment design.

Ten weeks after the ovariectomy, on day 0, a cotton ligature (cotton thread #24; Coats Corrente, São Paulo, Brazil) was placed around the first lower left molars using modified forceps. Surgical knots [57] were used to keep the ligature in place for 14 days, allowing biofilm buildup to induce EP.

After this period, the ligature was removed (Figure 1b), and the animals were randomly distributed into four experimental groups: NLT (*n* = 14), SRP (*n* = 14), SRP-BGPee (*n* = 14), and SRP-BGPlns (*n* = 14). In the NLT group, no local treatment was performed. In the SRP group, scaling root planing and irrigation with physiological saline solution were performed. In the SRP-BGPee group, scaling root planing and irrigation with green propolis extract were performed. In the SRP-BGPlns group, scaling root planing and irrigation with BGPlns were performed. EE was aware of the group allocation at the stages of the experiment.

A single SRP session was performed. SRP was performed immediately after ligature removal with mini-five 1–2 manual curettes (Hu-Friedy Co. Inc., Chicago, IL, USA) through ten distomesial movements on the buccal and lingual surfaces and ten cervico-occlusal movements on the interproximal and furcation areas [57] (Figure 1c).

The first irrigation happened immediately after SRP and 2, 4, and 6 days after the procedure. The SRP group received irrigation with physiological saline solution, the SRP-BGPee group received irrigation with ethanolic extract of BGP (Figure 1d), and the SRP-BGPlns group received irrigation with BGPlns (Figure 1e). A syringe with a needle containing 1 mL of the respective solution was used to direct the solutions towards the gingival sulcus. After irrigation, the solutions were left in place for 5 min, and only excess solution was removed using sterile cotton. Precautions were taken to prevent the swallowing of the irrigating solutions. This included placing a cotton barrier at the bottom of the oral cavity and ensuring the positioning of the animal on the operating table prevented the solutions from flowing into the posterior portion of the oral cavity.

Seven days and twenty-eight days after the ligature removal, the rats were euthanized via transcardiac perfusion with 100 mL of 0.9% NaCl solution plus 0.1% heparin, followed by 800 mL of fixative solution consisting of 4% formaldehyde (Sigma-Aldrich, St. Loius, MO, USA) in phosphate-buffered saline (PBS, 0.1 M, 4 °C, pH 7.4). The left hemimandibles were dissected and subjected to post-fixation in the same fixative solution for 48 h.

### 2.4. Analysis of General Health Condition and Intraoral Examination

The general health of the animals and body weight were periodically assessed throughout the experimental period. After euthanasia, an intraoral examination was performed, which consisted of a thorough visual inspection of the oral cavity, especially the sites with EP.

### 2.5. Computerized Microtomography of Samples

The hemimandibles of animals euthanized on the 28th day twenty-eight days after the ligature removal were analyzed using the Micro-CT system (SkyScan 1272, Brucker, Billerica, MA, USA). The samples were scanned with an isotopic voxel size of 0.9 mm, 50 kVp, a rotation angle of 0.5°, beam current at 500μA, an aluminum filter of 0.5 mm, an exposure time of 540 milliseconds, and an image resolution of 8 μm. The NRECON^®^ software (software program version 1.6.3.3) was used to reconstruct the images of each sample. Linear measurements (coronal, sagittal, and transaxial axis) in all three spatial dimensions were visualized and evaluated using the DataViewer^®^ program (software version 1.5.0). Finally, all images were oriented and saved in coronal slices (2000 × 1336). To determine the central region of the furcation, a 3D ROI (region of interest) was selected using the CTAn^®^ software program version 1.10.11.0. An examiner (G.R.C.S.) used morphological landmarks to guide the ROI design until the mesial and distal pulp of the lower first molar was identified. After identification, 50 sections (25 anterior and 25 posterior) were selected from the central region of the furcation for microtomographic analysis. Volumetric measurements were then performed. The contours were drawn below the cementoenamel junction (CEJ). They extended 1 mm towards the apical direction to ensure that the entire central area of the furcation region of the first molar was included in the ROI. The bone volume/bone total in the furcation region (BV/TV-%) was measured. To analyze the level of alveolar bone loss using the DataViewer^®^ program, four linear measurements were performed, from the cementoenamel junction to the alveolar bone crest (CEJ-ABC) on the vestibular, lingual, and distal surfaces of the tooth and one in the center of the furcation region of the external surface of the cementum to the alveolar bone crest.

### 2.6. Histological and Immunohistochemical Processing of Samples

The samples were washed in water and immersed in a demineralizing solution of 10% ethylenediaminetetraacetic acid (EDTA; Sigma-Aldrich) in PBS for 60 days. After demineralization, the samples were washed in water, dehydrated in ethanol, diaphanized in xylene, impregnated and embedded in histological paraffin, and sectioned with a microtome at 5 µm thickness. The microtomy was performed following a cutting plane that progressed from vestibular to lingual of the first lower left molar with EP. The histological sections were subjected to hematoxylin-eosin (HE) staining for histopathological analysis of the periodontal tissues and histometric analysis of the percentage of bone tissue in the furcation region (PBT) of the first lower molar with EP.

The histological sections underwent deparaffinization in a histology oven at a temperature of 56 °C to 60 °C for 30 min. After this, the sections were immersed in three separate baths of xylene for 10 min each (I, II, III). Rehydration was carried out through baths in ethanol, followed by incremental volumes of water at temperatures of 100 °C, 95 °C, and 70 °C, with each bath lasting 2 min. After this process, as well as after each stage of the immunohistochemical reaction, the sections were washed with PBS, PBS-TX, and PBS for 5 min. For antigen recovery, the histological sections were immersed in a citrate buffer within a pressurized chamber at 95 °C for 10 min. The sections prepared for the detection of osteocalcin (OCN) and transforming growth factor beta 1 (TGFβ1) were subjected to the indirect immunoperoxidase technique. The sections intended for detecting tartrate-resistant acid phosphatase (TRAP)-positive cells were processed using the indirect immunofluorescence technique.

For the indirect immunoperoxidase technique, endogenous peroxidase and nonspecific sites were blocked with 3% hydrogen peroxide for 1 h and 1.5% bovine serum albumin for 12 h, respectively. The histological sections were divided into two batches, which were incubated for 24 h with the following primary antibodies: mouse anti-TGF-β1 (sc376875, 1:200; Santa Cruz Biotechnology, Dallas, TX, USA) and mouse anti-OCN (sc365797, 1:100; Santa Cruz Biotechnology, Dallas, TX, USA). For signal amplification, a horse anti-mouse/rabbit IgG biotinylated antibody (BA-1400, 1:100; Vector Laboratories, Newark, CA, USA) was used for 2 h, and streptavidin conjugated with HRP (SA-5004, 1:100; Vector Laboratories) for 2 h. Development was performed using 3,3’-diaminobenzidine tetrahydrochloride (DAB) (SK-4100, ImmPACT DAB Substrate kit, peroxidase, Vector Laboratories, Newark, CA, USA) as the chromogen for 1 min [58]. No counterstaining was performed. Finally, the histological sections were dehydrated in ethanol, diaphanized in xylene, and protected by mounting with a glass coverslip. As a negative control for this type of immunohistochemical reaction, the specimens were subjected to the abovementioned procedures, suppressing the use of primary antibodies.

For the indirect immunofluorescence technique, nonspecific sites were blocked with 1.5% bovine serum albumin for 12 h. The histological sections were incubated for 24 h with a mouse anti-TRAP antibody (sc376875, 1:200; Santa Cruz Biotechnology, Dallas, TX, USA). For signal amplification, donkey anti-mouse IgG conjugated with Alexa Fluor 488^®^ (ab150129, ABcam, Cambridge, UK) was used for 2 h. The nuclei were counterstained with DAPI (D9542, Sigma Aldrich, St. Loius, MO, USA). Finally, the histological sections were protected by a glycerol-based mount and glass coverslip. As a negative control for this type of immunohistochemical reaction, the specimens were subjected to the abovementioned procedures, without the use of the primary antibody.

### 2.7. Histopathological and Histometric Analysis of the PBT Analyses

Histopathological analyses were performed by a certified histologist (E.E.) blinded to the treatments. The following parameters were evaluated in the first lower molar: intensity of the local inflammatory response, extent of the inflammatory process, pattern of connective tissue structuring in the furcation, and pattern of bone tissue structuring in the furcation region. Our research group established the parameters used in the histopathological analysis in a previous study [57].

Images of the furcation region of the first lower molar, with 25× magnification, were captured using a digital camera (AxioCam^®^, Carl Zeiss, Gottingen, Germany) connected to an optical microscope (AxioLab^®^, Carl Zeiss, Gottingen, Germany) and linked to a computer. We employed three equidistant histological sections to accurately determine the Percentage of Bone Tissue in the Furcation Region (PBT). The image analysis program Axiovision 4.8.2^®^ (Carl Zeiss, Gottingen, Germany) was used to determine the PBT. Initially, the Total Furcation Area (TFA) was measured, followed by the Area Occupied by Bone Tissue (ABT), both in mm^2^. The apical border of the TFA was determined by drawing a straight line from the apex of the mesial root to the apex of the distal root in order to calculate the PBT. Then, the entire contour of the external surface of the cementum located between the roots was followed. The same apical limit of the TFA was used to measure the ABT, and the contour of the external surface of the bone tissue between the roots was followed. The PBT was calculated by multiplying ABT by 100 and dividing by TFA (PBTF = (ABT × 100)/TFA [57]. The PBT was expressed as a mean percentage ± standard deviation.

### 2.8. Immunolabeling Pattern of TRAP, TGF-β1, and OCN in the Furcation Region of the First Lower Molar

Images were obtained at a magnification of 200× from the furcation area of the first lower molar. A square with sides measuring 2000 µm was positioned in the center of the interradicular septum in the acquired images. For TGF-β1, the coronal limit of this square was defined as the external surface of the cementum at the base of the furcation region, extending apically for a distance of 2000 µm. For OCN and TRAP, the coronal limit of this square was the alveolar bone crest in the furcation region, from which it extended apically for a distance of 2000 µm.

Immunolabeling analysis for TGF-β1 and OCN was performed using the Color Threshold tool in the ImageJ^®^ program (version 1.5i; National Institute of Health), where the immunolabeling density was obtained [57,58]. Immunolabeling for TGF-β1 and OCN was expressed as a percentage of the immunolabeling area ± standard deviation in each experimental group.

For the TRAP analysis, the number of immunolabeling multinucleated cells was counted [58]. The quantity of TRAP-positive cells was reported as the number of cells per mm^2^ ± standard deviation for each experimental group.

### 2.9. Statistical Analysis

PBT was considered the primary outcome. Microtomographic analysis, histopathological analysis, and immunolabeling for TRAP, TGFβ1, and OCN were considered secondary outcomes. Bioestat 5.3 software (https://www.mamiraua.org.br/pt-br/downloads/programas/bioestat-versao-53; Instituto Mamiruá, Manaus, Brazil, accessed on 10 May 2024) was used for statistical analyses. The Shapiro–Wilk test was used to analyze the distribution of data related to ECJ-ABC; BV/TV; PBT; and immunolabeling for TRAP, TGFβ1, and OCN. Considering that all variables evaluated presented a normal distribution, Analysis of Variance (ANOVA) followed by Tukey’s post hoc test was used. For the histopathological analysis, the Analysis of Variance of the Kruskal–Wallis and the Student–Newman–Keuls post hoc test were used. The significance level adopted in all statistical tests was 5% (*p* < 0.05).

## 3. Results

### 3.1. Characterization of the Physicochemical Properties of Lipid-Based Nanostructures Containing BGP

**Figure 2:**
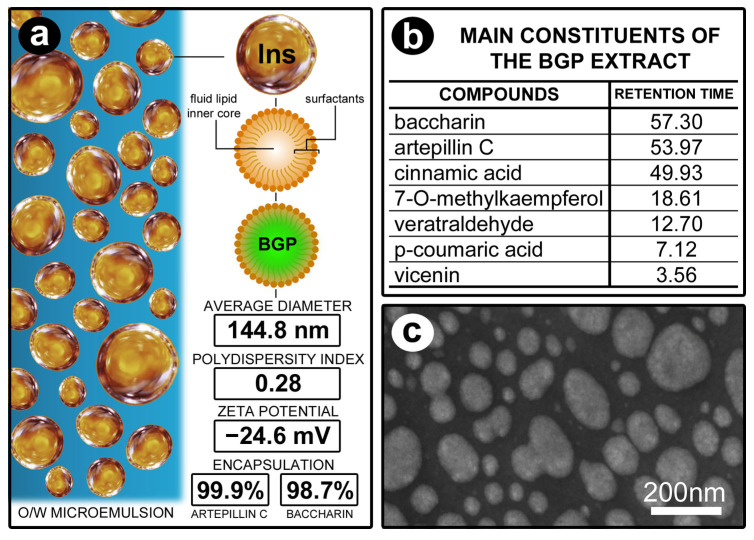
BGP-loaded lipid nanostructure (BGPlns). (**a**) Schematic illustration of BGPlns. (**b**) Table containing the major phytochemical compounds identified in the BGP extract by HPLC-DAD (λ = 275 nm). (**c**) Electron micrograph showing the appearance of BGPlns after 90 days of preparation (storage at 25 °C). Scale bars: 200 nm.

The lipid-based nanostructures (O/W microemulsion) containing BGP had a spherical morphology, an average diameter of 144.8 nm, a polydispersity index of 0.28, and a zeta potential of −24.6 mV. The encapsulation efficiency was 99.9% for artepillin C and 98.7% for baccharin. Figure 2 illustrates the main characteristics of BGPlns, shows the main phytochemical compounds identified in the BGP extract, and presents the BGPlns visualized in transmission electron microscopy after 90 days of preparation (storage at 25 °C) (Figure 2).

### 3.2. ECJ–ABC

**Figure 3:**
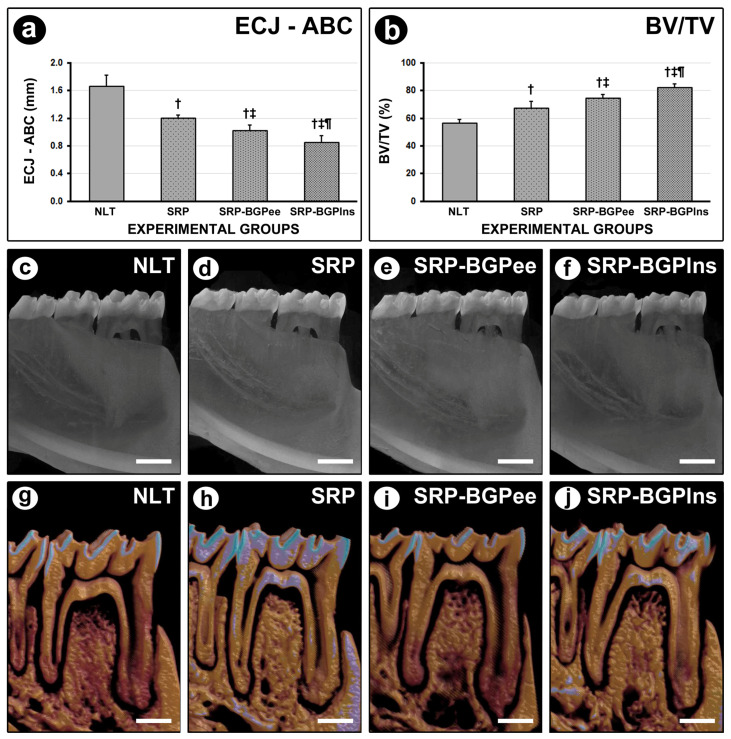
Microtomographic appearance of the lower first molar at 28 days. (**a**,**b**) Graphs showing the mean linear distance from the cementoenamel junction to the alveolar bone crest (CEJ-ABC) (**a**) and Bone Volume/Total Volume (BV/TV) in the furcation region at 28 days. Statistical tests: Analysis of Variance (ANOVA) and Tukey post hoc test. (**c**–**j**) Microtomographic representative images of NLT (**c**,**g**), SRP (**d**,**h**), SRP-BGPee (**e**,**i**), and SRP-BGPlns (**f**,**j**) groups. Symbols: †, statistically significant difference compared to the NLT group; ‡, statistically significant difference compared to the SPR group; ¶, statistically significant difference compared to the SPR-BGPee group. Scale bars: 1 mm.

The mean distance between the ECJ and ABC was lower in the SRP-BGPlns group when compared to the other experimental groups. The SRP-BGPee group had a lower mean distance between the ECJ and ABC than the NLT and SRP groups. In the SRP group, the mean distance between the ECJ and ABC was lower than in the NLT group (Figure 3).

### 3.3. BV/TV in the Furcation Region of the First Lower Molar

The SRP-BGPlns group presented higher BV/TV when compared to the other experimental groups. The BV/TV in the SRP-BGPee group was higher than in the NLT and SRP groups. In the SRP group, the BV/TV was higher than in the NLT group (Figure 3).

### 3.4. PBT in the Furcation Region

**Figure 4:**
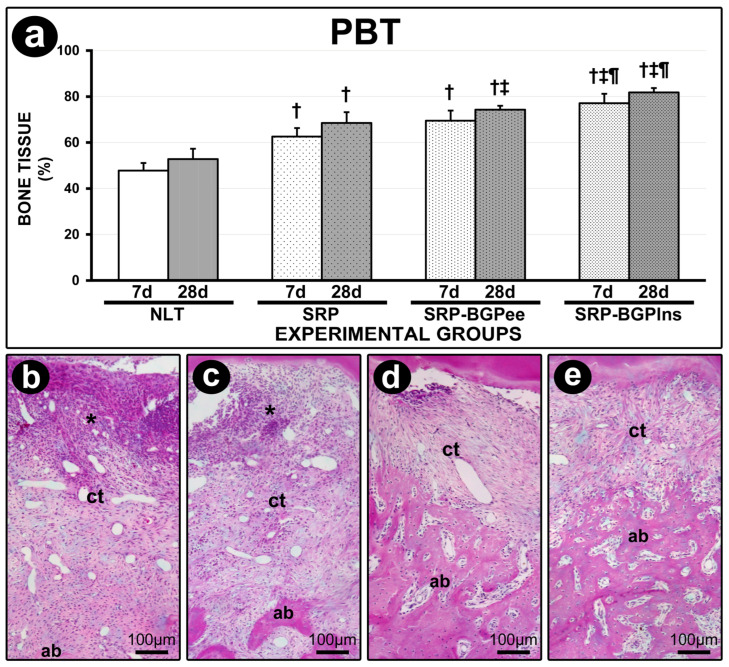
Bone tissue in the furcation region of the lower first molar. (**a**) Graph showing the percentage of bone tissue in the furcation region (PBT) in the different experimental groups. Statistical tests: Analysis of Variance (ANOVA) and Tukey post hoc test. (**b**–**e**) Photomicrographs showing the histological aspect of the furcation region of the lower first molar in the NLT (**b**), SPR (**c**), SPR-BGPee (**d**), and SRP-BGPlns (**e**) groups at 7 days. Abbreviations and symbols: *, inflammatory infiltrate; ct, connective tissue; ab, alveolar bone; †, statistically significant difference compared to the NLT group in the same period; ‡, statistically significant difference compared to the SPR group in the same period; ¶, statistically significant difference compared to the SPR-BGPee group in the same period. Staining: Hematoxylin and Eosin (H&E). Original magnification: (**b**–**e**): 200×. Scale bars: (**b**–**e**): 100 μm.

The SRP-BGPlns group showed higher PBT when compared to the other experimental groups at 7 days and 28 days. The PBT in the SRP-BGPee group was higher than in the NLT and SRP groups both at 7 days and 28 days. In the SRP group, the PBT was higher than in the NLT group at 7 days and 28 days (Figure 4).

### 3.5. Histological Analysis of Periodontal Tissues

The parameters, scores, and distribution of specimens according to histopathological analysis in the NLT, SRP, SRP-BGPee, and SRP-BGPlns groups are presented in Table 1. The SPR-BGPlns group showed the most favorable histological characteristics. Seven days after the procedure, this group exhibited a lower magnitude of the inflammatory response and less involvement of both connective and bone tissues. By 28 days post-operatively, the local inflammatory process had progressed toward resolution, and both the connective and bone tissues in the area were well structured. (Table 1 and Figure 4).

### 3.6. TRAP Immunolabeling

**Figure 5:**
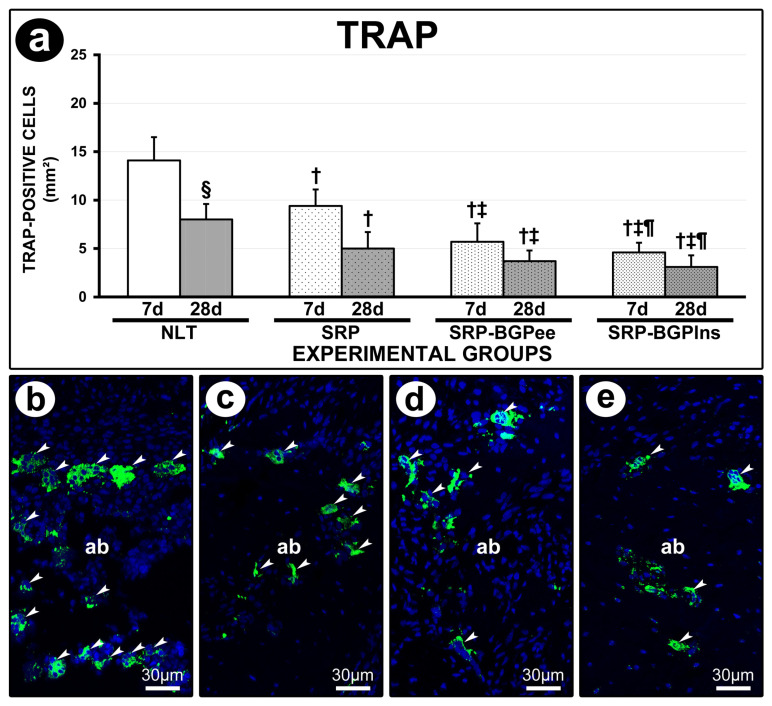
TRAP immunolabeling in the furcation region of the lower first molar. (**a**) Graph showing the number of TRAP-positive cells in the different experimental groups. Statistical tests: Analysis of Variance (ANOVA) and Tukey post hoc test. (**b**–**e**) Photomicrographs showing the immunolabeling pattern for TRAP in the furcation region of the lower first molar in the NLT (**b**), SPR (**c**), SPR-BGPee (**d**), and SRP-BGPlns (**e**) groups at 7 days. Abbreviations and symbols: ab, alveolar bone; white arrows, TRAP-positive cells; †, statistically significant difference compared to the NLT group in the same period; ‡, statistically significant difference compared to the SPR group in the same period; ¶, statistically significant difference compared to the SPR-BGPee group in the same period; §, statistically significant difference compared to 7 days in the same group. Original magnification: (**b**–**e**): 400×. Scale bars: (**b**–**e**): 30 μm.

At both 7 and 28 days, the NLT group exhibited a higher number of TRAP-positive cells compared to the other experimental groups. At 7 days, the SRP-BGPee and SRP-BGPlns groups showed a lower number of TRAP-positive cells than the SRP group. Additionally, in both the NLT and SRP groups, the count of TRAP-positive cells was lower at 28 days compared to 7 days (Figure 5).

### 3.7. TGFβ1 Immunolabeling

**Figure 6:**
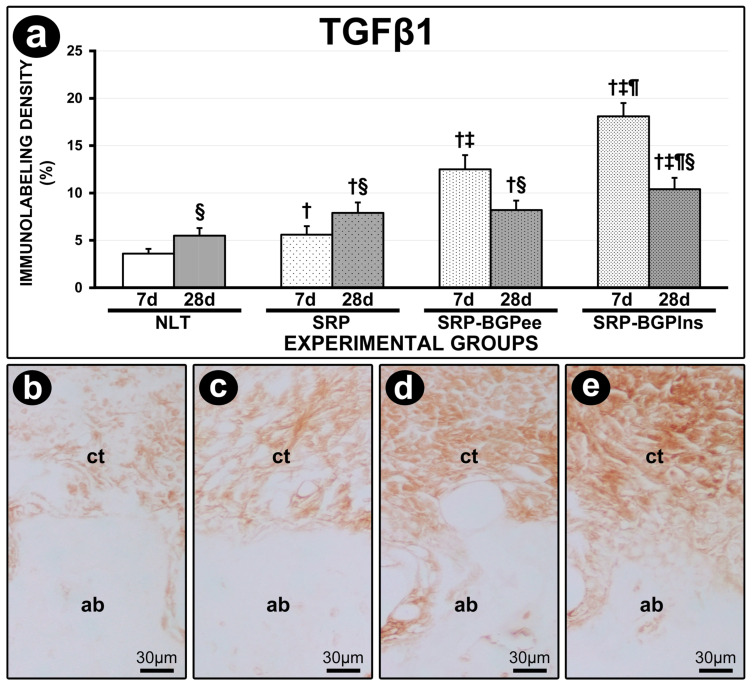
TGFβ1 immunolabeling in the furcation region of the mandibular first molar. (**a**) Graph showing the density of immunolabeling for TGFβ1 in the different experimental groups. Statistical tests: Analysis of Variance (ANOVA) and Tukey post hoc test. (**b**–**e**) Photomicrographs showing the immunolabeling pattern for TGFβ1 in the furcation region of the mandibular first molar in the NLT (**b**), SPR (**c**), SPR-BGPee (**d**), and SRP-BGPlns (**e**) groups at 7 days. Abbreviations and symbols: ct, connective tissue; ab, alveolar bone; †, statistically significant difference in relation to the NLT group in the same period; ‡, statistically significant difference compared to the SPR group in the same period; ¶, statistically significant difference compared to the SPR-BGPee group in the same period; §, statistically significant difference compared to 7 days in the same group. Original magnification: (**b**–**e**): 400×. Scale bars: (**b**–**e**): 30 μm.

The immunolabeling density for TGFβ1 in the furcation region of the lower first molar in the SRP-BGPlns group was higher than in the other experimental groups both at 7 days and 28 days. At 7 days, the SRP-BGPee group exhibited a higher immunolabeling density for TGFβ1 compared to the SRP group. In both experimental periods, the SRP and SRP-BGPee groups showed a higher immunolabeling density for TGFβ1 compared to the NLT group. The immunolabeling density for TGFβ1 in the NLT and SRP groups was higher at 28 days compared with 7 days; in contrast, in the SRP-BGPee and SRP-BGPlns groups, it was lower at 28 days compared with 7 days (Figure 6).

### 3.8. OCN Immunolabeling

**Figure 7:**
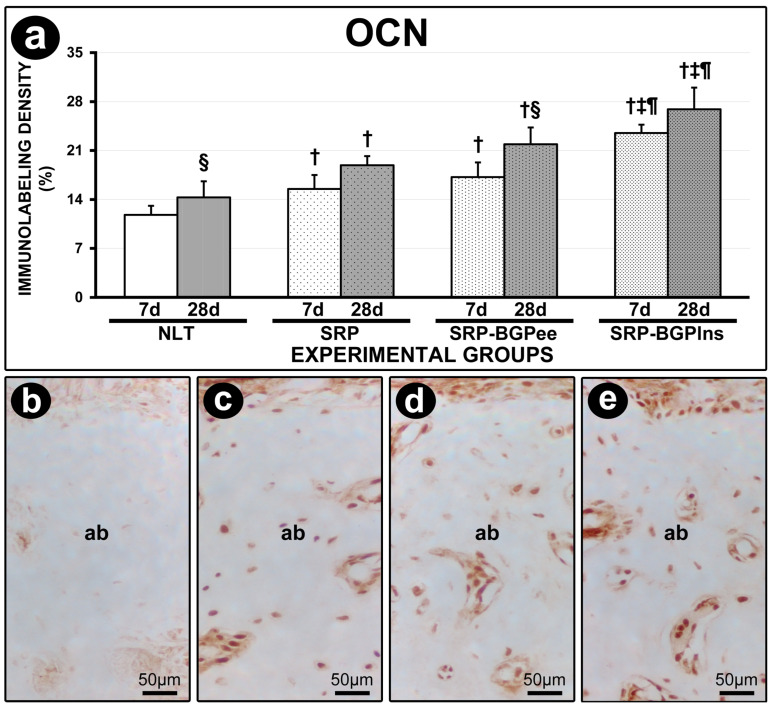
OCN immunolabeling in the furcation region of the lower first molar. (**a**) Graph showing the density of immunolabeling of OCN in the different experimental groups. Statistical tests: Analysis of Variance (ANOVA) and Tukey post hoc test. (**b**–**e**) Photomicrographs showing the pattern of immunolabeling for OCN in the furcation region of the lower first molar in the NLT (**b**), SPR (**c**), SPR-BGPee (**d**), and SRP-BGPlns (**e**) groups at 28 days. Abbreviations and symbols: ab, alveolar bone; †, statistically significant difference compared to the NLT group in the same period; ‡, statistically significant difference compared to the SPR group in the same period; ¶, statistically significant difference compared to the SPR-BGPee group in the same period; §, statistically significant difference compared to 7 days in the same group. Original magnification: (**b**–**e**): 400×. Scale bars: (**b**–**e**): 50 μm.

The immunolabeling density for OCN in the furcation region of the lower first molar in the SRP-BGPlns group was higher than in the other experimental groups both at 7 days and at 28 days. In both experimental periods, the SRP and SRP-BGPee groups presented higher immunolabeling density for OCN compared to the NLT group. The immunolabeling density for OCN in the SRP-BGPee group was higher at 28 days compared to 7 days (Figure 7).

## 4. Discussion

Periodontitis has a multifactorial etiology, involving complex interactions among genetic, microbiological, and immunological factors [1,2,3]. In cases of estrogen deficiency, the severity of periodontitis increases, as evidenced by both animal experimental studies and clinical studies on humans [7,8,9,10,11,12,13,14,15,16]. The gold standard treatment for periodontitis is SRP [17,18,19]. While this method is effective, it has limitations, in some regions and in certain clinical situations, mainly when severe tissue destructuring occurs in the host and the response of periodontal tissues to treatment presents some degree of impairment. In these cases, the use of adjuvant therapies aid in achieving the necessary effectiveness for controlling or managing periodontitis [20,21]. Although some studies have already shown the positive effects of ethanolic extract of propolis as an adjuvant therapy to SPR in the management of periodontitis [37,38,39,40,41,42], the use of BGP incorporated into lipid-based nanostructures has been studied only by our research group. Lipid-based nanostructures such as O/W microemulsion can enhance the physicochemical properties of the active ingredients, thereby improving the biological effects of these substances [48,49,50,51]. This study aimed to evaluate the effects of local application of BGP, either as an ethanolic extract or incorporated into lipid-based nanostructures, as an adjuvant therapy to SRP in the management of EP in ovariectomized rats. The null hypothesis of this study posits that SRP combined with the local application of BGP, whether in the form of an ethanolic extract or incorporated into lipid-based nanostructures, does not provide greater effectiveness compared to conventional mechanical treatment of EP in ovariectomized rats. Our findings indicate that SRP associated with irrigation using BGP was effective in treating EP. Specifically, irrigation with BGPlns was more effective than irrigation with the BGPee. Consequently, our findings refute the null hypothesis.

The current study utilized an experimental model of ligature-induced periodontitis in ovariectomized rats. Some studies have indicated that the extent of the local inflammatory response and the degree of alveolar bone loss are greater in ovariectomized rats with periodontitis compared to controls [7,8,9,10,11]. It has been demonstrated that ovariectomized rats with EP subjected to SRP exhibited greater alveolar bone loss and increased immunolabeling for TRAP and RANKL in the periodontium compared to sham-operated rats, indicating that the response to conventional mechanical treatment is deficient following ovariectomy [59]. This experimental model was used in this study because it presents a more challenging condition for the organism, characterized by a more pronounced progression of the disease and a limited response to treatment. In these circumstances, the use of adjuvant therapies in conjunction with SRP can help achieve better treatment outcomes. Our focus was on the furcation region due to its anatomical features that promote bacterial proliferation, restrict access for periodontal instruments, and represent a niche that could significantly benefit from the local adjuvant therapy employed in this study.

The use of nanoformulations can address certain limitations associated with natural active ingredients, such as BGP, thereby significantly enhancing their effectiveness. In this study, BGP was incorporated into an O/W microemulsion, a lipid-based nanostructure. This type of nanostructure enhances solubility, increases bioavailability, facilitates interaction and passage through biological membranes, protects against rapid degradation, and prolongs the effects of the active ingredient [52,53,54,55]. It has been showed that nanoencapsulated green propolis presented the same analgesic effect and anti-inflammatory action when used in a concentration 10 times lower than free green propolis, which denotes the enhancement of the biological effects of nanoformulations [60].

Our results showed that SRP combined with irrigation with both the ethanolic extract of BGP and BGPlns reduced periodontal inflammation. Notably, the most significant effects were observed in the SRP-BGPlns group. These findings support previous studies that have reported the ability of the propolis, whether in aqueous or ethanolic extract form, to alleviate inflammation in periodontal tissues [34,35,36,37,38,39,40,41,42]. BGP has several bioactive compounds with anti-inflammatory action, of which artepillin C [30,61,62] and baccharin [63,64] stand out. Propolis has been shown to regulate inflammasomes and inflammatory genes, which in turn reduces the levels of cytokines and chemokines with proinflammatory activity [65]. Lipid-based nanostructures significantly improve the physicochemical properties of natural active ingredients, such as BGP. This enhancement increases their biological effects, including their anti-inflammatory action [60], which helps explain the improved results observed in SRP-BGPlns group.

By modulating the local inflammatory response and reducing levels of proinflammatory cytokines, the levels of RANK, an important cytokine that regulates the formation and activity of osteoclasts, are also decreased [66]. This reduction may prevent the activation of RANK, which is present in both osteoclast precursors and mature osteoclasts [66]. Furthermore, propolis is capable of increasing the levels of OPG [67], a decoy receptor for RANKL, which inhibits RANK activation [66]. The local effects include reduced osteoclastogenesis and decreased resorptive activity of mature osteoclasts, leading to decreased alveolar bone loss. Upon analyzing the number of TRAP-positive cells in the furcation region, we found that SRP associated with the irrigation of BGP, ethanolic extract and carried in lipid-based nanostructures, reduced the recruitment of osteoclasts. This effect was particularly pronounced at seven days post-operatively, a time when the number of osteoclasts is typically higher. Furthermore, when we evaluated Bone Volume/Total Volume and PBT in the furcation region, we observed that the SRP-BGPlns group presented a greater amount of bone tissue. This suggests that the treatment with BGPlns resulted in a more substantial reduction in osteoclastic activity, thereby contributing to the better preservation of alveolar bone tissue in this experimental group.

SRP associated with the irrigation of BGP (ethanolic extract and carried in lipid-based nanostructures) facilitated the resolution of inflammation and accelerated the progression to tissue repair. Propolis has been shown to accelerate and improve tissue repair by stimulating key cells involved in the process, such as endothelial cells, keratinocytes, fibroblasts, and osteoblasts [67,68,69,70,71,72]. TGFβ1 is a cytokine that plays an essential role in limiting and resolving inflammation, as well as regulating the proliferation, differentiation, and activity of various cell types [73,74,75]. Its levels are frequently used to evaluate the progression of periodontal treatment [76,77]. In the present study, we observed a higher density of TGFβ1 immunolabeling at seven days post-operative in the groups treated with SRP associated with the irrigation of BGP. Notably, the BGPlns group exhibited the highest level of TGFβ1 immunolabeling. The findings related to TGFβ1 immunolabeling cannot be interpreted in isolation. When examined in association with the other analyses performed in the present study, especially the histopathological analysis of connective and bone tissues, we found that the greater immunolabeling to TGFβ1 at 7 days post-operatively is probably related to the increase in both the proliferation and differentiation of cells responsible for the structuring of such tissues; that is, there was an acceleration of the tissue repair process. Consequently, at 28 days post-operatively, the periodontal tissues were much better structured in the groups treated with BGP, especially in the SRP-BGPlns group.

The SRP-BGPlns group exhibited significant changes in the connective tissue of the furcation region. At 7 days post-operatively, the cellularity and structure of the extracellular matrix indicated an accelerated repair process. At 28 days post-operatively, the quality of this tissue had improved significantly; it developed into dense connective tissue with a histological appearance very similar to that of the periodontal ligament found in healthy conditions, which had never been impacted by periodontal disease. Our results also show that the use of SRP associated with the irrigation of BGP-loaded lipid nanostructure delivered via lipid nanoparticles induced local bone neoformation. Histopathological analysis conducted 28 days post-treatment revealed that the SRP-BGPlns group exhibited thin bone trabeculae filled with many osteocytes and surrounded by active osteoblasts. These bone trabeculae originated from the more mature bone tissue located in the interradicular septum of the furcation region. The observed pattern of cellularity and the structure of the extracellular matrix suggest that these bone trabeculae represent newly formed bone tissue. Research indicates that propolis can activate transcription factors that regulate osteoblast differentiation and positively influence growth factors that promote both angiogenesis and local osteogenesis [67,71,72].

Although the antimicrobial effects of BGP were not the primary focus of our study, it is an important aspect to consider regarding its local effects. The antimicrobial properties of propolis are predominantly attributed to flavonoids [29,78]. It has been reported that propolis, in either aqueous or ethanolic extract form, is effective against both Gram-positive and Gram-negative bacteria, with the latter being less sensitive to these formulations. One possible explanation for the lower effectiveness against Gram-negative bacteria is the protective role of the complex lipid content in their cell walls [79]. However, in the SRP-BGPlns group, the BGP was carried in lipid-based nanostructures, which may easily overcome this barrier. As a result, this formulation could enhance the antimicrobial effect even against Gram-negative bacteria. In addition, studies have also reported that BGP extract has antimicrobial and antibiofilm action on several bacteria, including key periodontopathogens [32,80,81]. It has been reported that that the ethanolic extract of propolis and some of its isolated compounds, artepillin C, baccharin, and ursolic acid, were effective against *Porphyromonas gingivalis*. Through different mechanisms, the extract and its isolated components mainly caused structural and functional damage to the bacterial membrane. This study also showed a potent bactericidal effect of ursolic acid, mainly attributed to its highly lipophilic nature [82]. This last finding corroborates that BGP carried in lipid-based nanostructures ensures greater effectiveness even for components that do not have this lipophilic nature. Once the antimicrobial action is exerted, particularly in challenging areas for periodontal instruments, such as the furcation region, propolis contributes to enhanced treatment effectiveness. Control of local microbiota contributes to reducing inflammation and creating an environment conducive to tissue repair.

In general, the findings of the present study corroborate a previous study developed by our research group. It was used an experimental model of EP in ovariectomized rats undergoing therapy with a high dosage of zoledronate, i.e., at risk for medication-related osteonecrosis of the jaw (MRONJ). The study showed that EP without treatment, or treated exclusively with SRP, resulted in reduced alveolar bone loss, given the inhibition of the resorptive activity caused by zoledronate. However, they caused an exacerbated inflammatory response and a significant increase in the amount of non-vital bone tissue, significantly increasing the risk of MRONJ. When SRP was associated with irrigation with BGPlns, there was control of local inflammation and containment of bone necrosis, i.e., proving to be an effective and safe therapy in the management of EP during treatment with a potent antiresorptive drug, including being able to prevent MRONJ. In the present study, no antiresorptive drug therapy was established, i.e., osteoclast activity remained unchanged, and its modulation was related to EP and/or the treatments instituted. In this situation, when SRP was used in association with irrigation with BGPlns in the management of PE, significant control of inflammation, improvement in the structuring pattern of connective and bone tissue, and significant reduction in alveolar bone loss via greater preservation and/or neoformation of alveolar bone tissue were also observed [57].

Systematic reviews have shown that patients with periodontitis who received non-surgical periodontal treatment associated with local use of propolis extract showed significant improvement in some clinical parameters, such as reduced probing depth and clinical attachment gain, compared to the control groups [83,84,85,86]. The high scientific evidence from these studies clearly demonstrates the beneficial effects of propolis, in the form of an extract, as an adjunctive therapy to SRP in the management of periodontitis. Our findings in the SRP-GPee group corroborate those of these studies. The primary significance of this experimental group in the current study is to facilitate a direct comparison between green propolis, both in its commonly used ethanolic extract form and when incorporated into lipid-based nanostructures. Our study demonstrated that the lipid-based nanostructures (O/W microemulsion) used was even more effective in the evaluated context, enhancing the beneficial effects of green propolis. It is important to emphasize that this formulation has an excellent cost–benefit ratio. The manufacture of green propolis microemulsion does not require equipment, techniques, or reagents that substantially increase the production cost. It is one of the most economical lipid-based nanostructures available.

BGP is a complex mixture, with hundreds of active substances acting through different mechanisms that offer several benefits simultaneously, including anti-inflammatory, immunomodulatory, antioxidant, and antimicrobial effects. Its potential use as an adjunct therapy for managing periodontitis is particularly noteworthy, as this condition is influenced by the local microbiota and the host’s immune–inflammatory response and ability to repair tissue. Our findings showed that the association of SPR with the local use of BGP, carried in lipid-based nanostructures, ensured an effective treatment for EP, even superior to the use of BGP in the form of an ethanolic extract. This indicates that lipid-based nanostructures serve as an effective delivery system for BGP, likely due to their ability to enhance its properties and maximize its effects. It is also important to note that this study was conducted on animals, aiming to replicate conditions that occur in humans. Therefore, the findings should be regarded as valuable guidance for future research. Controlled and randomized clinical studies in humans are essential to assess the effectiveness of using irrigation with BGP-loaded lipid nanostructures as an adjunct to SRP in the treatment of periodontitis.

## 5. Conclusions

Irrigation using BGP is effective as an adjuvant therapy for non-surgical periodontal treatment in managing EP in ovariectomized rats. Irrigation with BGP-loaded lipid nanostructures is more effective than irrigation with the ethanolic extract of BGP. This suggests that the lipid-based nanostructure enhances the therapeutic effects of BGP as an adjunct to SRP.

## Figures and Tables

**Table 1:** The parameters, scores, and distribution of the specimens based on the histopathological analyses of the furcation region of the mandibular first molar in the NLT, SRP, SRP-BGPee, and SRP-BGPlns experimental groups.

Histopathological Analyses								
Parameters and Scores	Number of Samples							
	Experimental Groups							
	NLT		SRP		SRP-BGPee		SRP-BGPlns	
	7 d	28 d	7 d	28 d	7 d	28 d	7 d	28 d
**Intensity of Local Inflammatory Response**								
**(1)** absence of inflammation	-	-	-	-	1	3	2	6
**(2)** small quantity of inflammatory cells (less than 1/3 of cells were inflammatory cells)	-	-	-	5	4	4	5	1
**(3)** moderate quantity of inflammatory cells (1/3 to 2/3 were inflammatory cells)	1	4	6	2	2	-	-	-
**(4)** large quantity of inflammatory cells (more than 2/3 were inflammatory cells)	6	3	1	-	-	-	-	-
**Median**	**4**	**3**	**3**	**2 ^†^**	**2 ^†^**	**2 ^†^**	**2 ^†‡^**	**1 ^†‡^**
**Extension of Inflammatory Infiltrate**								
**(1)** absence of inflammation	-	-	-	-	1	3	2	6
**(2)** partial extension of connective tissue in the furcation region	-	-	-	7	5	4	5	1
**(3)** entire extension of connective tissue in the furcation region	3	4	6	-	1	-	-	-
**(4)** entire extension of connective tissue and bone tissue in the furcation region	4	3	1	-	-	-	-	-
**Median**	**4**	**3**	**3**	**2 ^†§^**	**2 ^†‡^**	**2 ^†^**	**2 ^†‡^**	**1 ^†^**
**Pattern of Structuration of the Connective Tissue in the Furcation Region**								
**(1)** moderate number of fibroblasts and large amount of collagen fibers (dense connective tissue)	-	-	-	-	-	3	2	6
**(2)** moderate amount of both fibroblasts and collagen fibers	-	-	-	6	3	4	5	1
**(3)** small amount of both fibroblasts and collagen fibers	3	6	6	1	4	-	-	-
**(4)** severe tissue disorganization with necrosis areas	4	1	1	-			-	-
**Median**	**4**	**3**	**3**	**2 ^†§^**	**3**	**2 ^†§^**	**2 ^†‡^**	**1 ^†‡^**
**Pattern of Structuration of the Alveolar Bone in the Furcation Region**								
**(1)** bone trabeculae with regular contours coated with active osteoblasts, including areas of new bone formation	-	-	-	1	-	1	-	7
**(2)** bone trabeculae with predominantly vital bone tissue, with few areas comprising non-vital bone tissue	-	-	-	6	4	6	7	-
**(3)** irregularly contoured bone trabeculae filled with active osteoclasts	7	7	7	-	3	-	-	-
**(4)** bone trabeculae filled with active osteoclasts and areas of necrotic bone	-	-	-	-	-	-	-	-
**Median**	**3**	**3**	**3**	**2 ^†^**	**2**	**2 ^†^**	**2 ^†‡^**	**1 ^†^**

Symbols: ^†^, statistically significant difference compared to the NLT group in the same period; ^‡^, statistically significant difference compared to the SPR group in the same period; ^§^, statistically significant difference compared to 7 days in the same group.

## Data Availability

The datasets used and/or analyzed during the current study are available from the corresponding author on reasonable request.
